# Case Report: Durable, well-tolerated complete response to epcoritamab plus pirtobrutinib in Richter transformation relapsing after CD19 CAR T-cell therapy

**DOI:** 10.3389/fmed.2026.1998014

**Published:** 2026-10-07

**Authors:** Ahmed Salleh Barefah

**Affiliations:** 1 Hematology Department, Faculty of Medicine, King Abdulaziz University Hospital, King Abdulaziz University, Jeddah, Saudi Arabia; 2 Hematology Research Unit, King Fahad Medical Research Center, King Abdulaziz University, Jeddah, Saudi Arabia

**Keywords:** bispecific antibody, Bruton tyrosine kinase inhibitor, CAR T-cell therapy, chronic lymphocytic leukemia, diffuse large B-cell lymphoma, epcoritamab, pirtobrutinib, Richter transformation

## Abstract

Richter transformation (RT) that relapses after CD19-directed chimeric antigen receptor (CAR) T-cell therapy has no established therapy and carries a dismal prognosis. We report an 84-year-old man with multiply relapsed, biopsy-proven diffuse large B-cell lymphoma-type RT and a high-risk MYC/BCL2 double-expressor phenotype who developed systemic relapse, with extensive osteolytic bone disease, after lisocabtagene maraleucel. Three cycles of atezolizumab produced no objective benefit. Treatment with epcoritamab, a CD3xCD20 bispecific antibody, plus pirtobrutinib, a non-covalent Bruton tyrosine kinase inhibitor, produced complete nodal metabolic resolution within 3 months that deepened to a complete metabolic response (Deauville score 2) by 10 treatment cycles, with resolution of two interim osteolytic lesions whose transient nature illustrates the interpretive challenges of PET/CT response assessment after cellular and T-cell-engaging therapies. The chemotherapy-free regimen was well tolerated at age 84, with no cytokine release syndrome, no neurotoxicity, and no cytopenias; the only toxicity was mild, reversible dexamethasone-related myopathy. The patient remains in ongoing clinical remission after approximately 10 months of therapy. This case extends the reported experience with epcoritamab plus pirtobrutinib specifically to the post-CAR T-cell relapse setting and suggests that this chemotherapy-free doublet may warrant consideration as a salvage strategy for older or frail patients with RT who are unfit for further cytotoxic therapy.

## 1 Introduction

Richter transformation (RT) remains among the most treatment-refractory complications of chronic lymphocytic leukemia (CLL) (1). Contemporary consensus recommendations emphasize histologic confirmation, early response assessment, and clinical-trial enrollment because no uniformly effective standard exists, particularly after failure of chemoimmunotherapy or cellular therapy (2). Although CD19-directed chimeric antigen receptor (CAR) T-cell therapy can induce durable remissions in selected patients with RT, post-CAR T-cell relapse is an increasingly important therapeutic challenge (3). We report sustained disease control with epcoritamab plus pirtobrutinib in an older patient with multiply relapsed, biopsy-proven diffuse large B-cell lymphoma (DLBCL)-type RT following lisocabtagene maraleucel (liso-cel).

## 2 Case description

An 84-year-old man was diagnosed with CLL in late 2022 and initially was asymptomatic and observed for approximately 6 months. The CLL showed unmutated IGHV and no detected TP53 mutation (4). As he became symptomatic, acalabrutinib was initiated in 2023. After 1 year of treatment, new headache and multifocal bone pain prompted fluorodeoxyglucose (FDG) PET/CT, which identified hypermetabolic osteolytic lesions suspicious for transformation. Core needle biopsy of a right anterior chest-wall rib lesion on September 30, 2024, demonstrated DLBCL-type RT. Mayo Clinic review showed a non-germinal-center B-cell phenotype with CD5, CD20, LEF1, partial CD23, PD-1/CD279, BCL2 (90%), BCL6 (30%), MUM1 (30%), and MYC (40%) expression. The Ki-67 proliferation index was approximately 30%. The lymphoma was a MYC/BCL2 double expresser (5), but fluorescence *in situ* hybridization showed neither MYC rearrangement nor MYC-IGH fusion.

At the time of RT diagnosis, the patient's relevant baseline comorbidities included hypertension, nonocclusive coronary artery disease, paroxysmal atrial fibrillation, type 2 diabetes, dyslipidemia, positive hepatitis b core antibody, benign prostatic hyperplasia and previous benign colonic polyps. No formal geriatric or frailty instrument (e.g., Comprehensive Geriatric Assessment, G8 screening, or Cumulative Illness Rating Scale-Geriatric) was administered; functional status was assessed clinically and documented as an Eastern Cooperative Oncology Group (ECOG) performance status of 0 with full independence in activities of daily living, which was maintained throughout the disease course described below. We note this distinction explicitly because ECOG performance status alone reflects functional status at a single time point and may not fully capture physiological reserve in an octogenarian patient.

Acalabrutinib was discontinued, and the patient received 2 cycles of R-mini-CHOP. Interim PET/CT demonstrated progressive disease. He subsequently received liso-cel (6) on February 17, 2025, with ibrutinib and achieved metabolic remission. On October 13, 2025, PET/CT documented systemic relapse with a right supraclavicular node measuring 3.2 × 1.9 cm (SUVmax, 23.2), an upper retrocaval nodal mass measuring 4.7 × 3.7 cm (SUVmax, 21.9), and numerous FDG-avid osteolytic lesions involving the skull, sternum, scapulae, ribs, vertebrae, pelvis, humeri, and femora, several with cortical erosion or fracture (Figure 1A). A right ischial biopsy confirmed recurrent DLBCL morphologically and immunophenotypically similar to the original RT. At relapse, the CD20-positive large B cells coexpressed CD5, BCL6 (60%), MUM1 (>90%), BCL2 (>90%), and MYC (40%–50%), remained CD10-negative, and had a Ki-67 index of approximately 90%, consistent with biologic evolution to a highly proliferative relapse.

**Figure 1:**
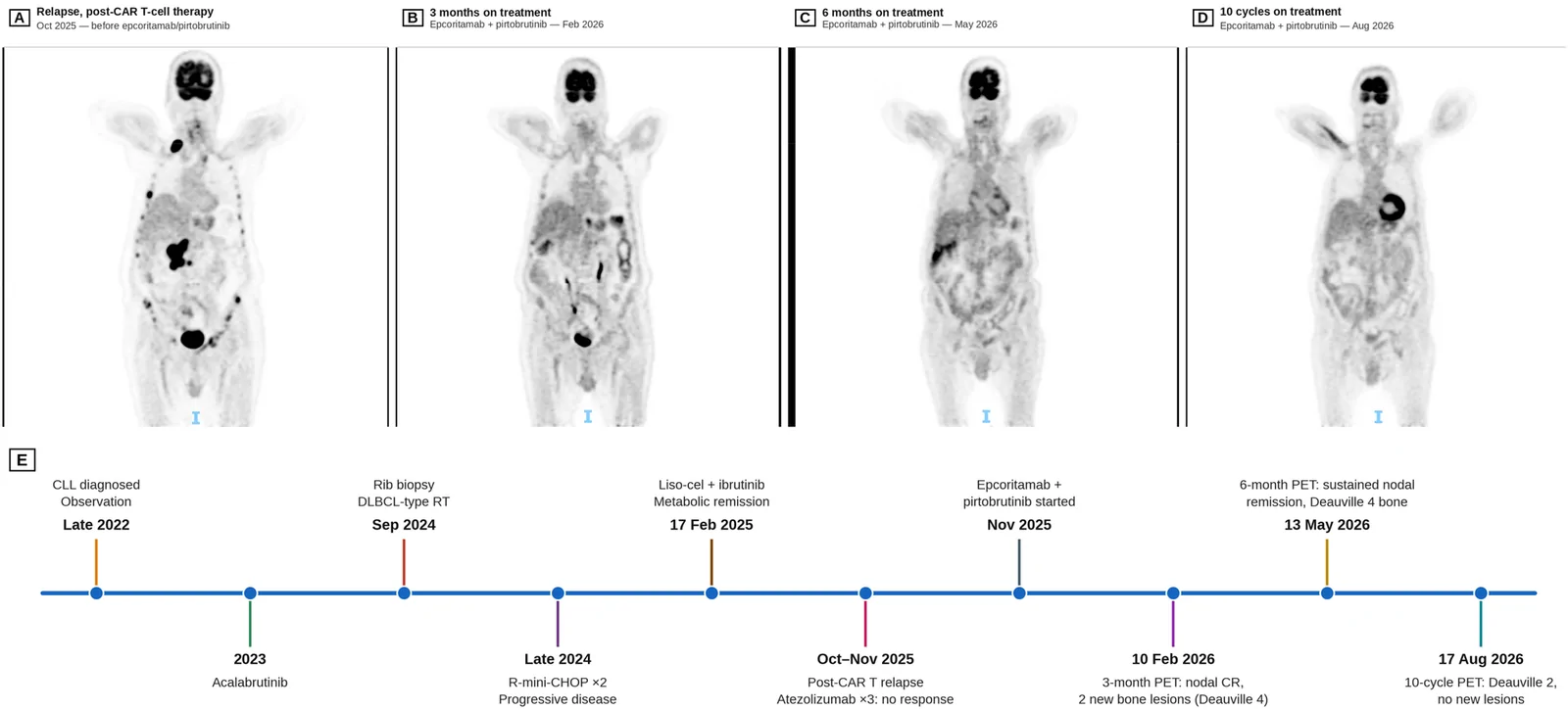
Disease course and metabolic response to epcoritamab plus pirtobrutinib. **(A)** Maximum-intensity-projection FDG-PET/CT on October 13, 2025, showing post-liso-cel relapse with intensely FDG-avid supra- and infradiaphragmatic lymphadenopathy (right supraclavicular node, SUVmax 23.2; upper retrocaval nodal mass, SUVmax 21.9) and widespread osseous involvement. **(B)** FDG-PET/CT on February 10, 2026, after approximately 3 months of epcoritamab plus pirtobrutinib, showing complete nodal metabolic resolution with marked but incomplete regression of skeletal uptake (Deauville score 4) and two new FDG-avid lytic lesions in the right humeral shaft and lower sacral segment. **(C)** FDG-PET/CT on May 13, 2026, after approximately 6 months of therapy, showing sustained nodal remission with residual Deauville score 4 uptake in the treated lytic lesions and no new lesions. **(D)** FDG-PET/CT on August 17, 2026, after 10 cycles of epcoritamab plus pirtobrutinib, showing resolution of the previously FDG-avid right humeral and sacral lesions and further regression of residual skeletal uptake to Deauville score 2, without new lesions. **(E)** Treatment timeline, which also serves as the required episode-of-care timeline for this case. PD, progressive disease; RT, Richter transformation. Panel-specific SUVmax values for the lesions in B–D are not available for annotation in the current figure files.

Atezolizumab was administered off-label as single-agent PD-L1 blockade at a standard dose of 1200 mg intravenously every 3 weeks reflecting reported signals of immune checkpoint inhibitor activity in RT, including as part of a combination regimen with venetoclax and obinutuzumab in the phase 2 MOLTO trial (7), and the absence of an accessible clinical trial for this patient at that time; here, atezolizumab was used off-label as single-agent therapy rather than as part of that combination. Three cycles produced no objective benefit and the agent was discontinued.

Because epcoritamab plus pirtobrutinib had no approved indication for RT at the time of treatment and no accessible clinical trial was available to this patient, the combination was administered as off-label therapy following institutional review and approval and a detailed discussion of the risks, uncertainties, and alternatives with the patient and his family, who provided informed consent. Epcoritamab plus pirtobrutinib was initiated in November 2025 with standard step-up dosing of 0.16 mg, 0.8 mg, then 48 mg subcutaneously according to the approved step-up schedule, together with continuous oral pirtobrutinib 200 mg once daily. The patient was hospitalized for observation around the first full (48 mg) dose per standard cytokine release syndrome (CRS)-mitigation protocol, including scheduled vital-sign monitoring, dexamethasone premedication, and bedside availability of tocilizumab and supportive medications; subsequent doses were administered in the outpatient setting.

An interim PET/CT on February 10, 2026, after approximately 3 months of therapy, already showed complete nodal metabolic resolution and normalized splenic uptake, with marked but incomplete regression of skeletal disease (Deauville score 4) and two new FDG-avid lytic lesions in the right humeral shaft and lower sacral segment, flagged for follow-up given uncertain neoplastic versus traumatic etiology (Figure 1B); this interim finding and its interpretation are discussed further below. PET/CT on May 13, 2026, after approximately 6 months of therapy, confirmed sustained nodal remission, with residual low-grade uptake in the treated lytic lesions and no new lesions (Deauville score 4; Figure 1C). Accordingly, the 6-month scan was conservatively classified as a partial metabolic response with complete nodal metabolic resolution rather than a complete metabolic response under Lugano criteria (8). A follow-up PET/CT on August 17, 2026, after 10 cycles of epcoritamab plus pirtobrutinib, showed further metabolic improvement, with resolution of the previously FDG-avid right humeral and sacral lesions and residual activity in the remaining osteolytic lesions now below mediastinal blood-pool uptake (Deauville score 2), without new lesions (Figure 1D); this scan is consistent with a complete metabolic response by Lugano criteria. The patient remains in ongoing clinical remission after approximately 10 months of therapy. The treatment course is summarized in Figure 1E.

Tolerability was a central consideration at age 84. No CRS occurred at any dose level, including the first full dose, and no immune effector cell-associated neurotoxicity syndrome was observed. No cytopenias developed, and neither growth-factor support nor transfusion was required.

Dexamethasone premedication was administered at a dose of 16 mg IV prior to each of the step-up doses per protocol and 16 mg orally for 3 days post each dose. Mild proximal myopathy was first noted approximately 3 weeks and was attributed to dexamethasone premedication; it prompted a reduction in the dexamethasone dose to 8 mg prior to each dose and omitting the post treatment dexamethasone, after which strength improved over 4 weeks and returned to baseline. Both epcoritamab and pirtobrutinib were continued throughout without interruption, dose reduction, or permanent discontinuation.

For infectious prophylaxis, he received monthly intravenous immunoglobulin (IVIg), valacyclovir 500 mg orally twice daily, and entecavir 0.5 mg orally daily for a positive hepatitis B core antibody. These measures reflected, respectively, humoral immunodeficiency related to prior anti-CD20 and cellular therapy (IVIg), standard herpesvirus prophylaxis during bispecific antibody and BTK-inhibitor therapy (valacyclovir), and guideline-directed prevention of hepatitis B reactivation in a patient with a positive hepatitis B core antibody receiving immunomodulatory therapy (entecavir); all three were continued throughout the treatment course described here. All the infectious prophylaxis are still continuing as the treatment is ongoing. The patient maintained an ECOG performance status of 0 and independent function throughout, and neither epcoritamab nor pirtobrutinib required permanent discontinuation.

## 3 Discussion

This response is notable given the patient's age, double-expressor phenotype, rapid increase in Ki-67, destructive osseous disease, and relapse after chemoimmunotherapy and CAR T-cell therapy. In the phase 1b/2 EPCORE CLL-1 study, epcoritamab monotherapy produced an overall response rate of 47.6% in 42 patients with RT after a median follow-up of 22.9 months; the overall response rate was 57.1% in first-line RT and 38.1% in second or later lines (9). This is consistent with epcoritamab's broader single-agent activity in relapsed or refractory large B-cell lymphoma (10). Separately, pirtobrutinib produced an overall response rate of 50% and a complete response rate of 13% in 82 patients with RT in the BRUIN study (11), building on established single-agent activity in heavily pretreated CLL more broadly (12). These data establish single-agent activity but do not determine the relative contribution of each drug when used together.

We note that the Ki-67 proliferation index of approximately 30% at initial RT diagnosis is lower than typically reported for DLBCL-type RT, which frequently exceeds 80% (1, 2). The diagnosis was nonetheless supported by concordant morphology, a non-germinal-center immunophenotype, and a MYC/BCL2 double-expressor profile on core needle biopsy, and was further corroborated by the marked increase in proliferative activity (Ki-67 ∼90%) and biologic evolution documented at relapse, consistent with an initially less proliferative RT clone that evolved toward a more aggressive phenotype.

Clinical experience with the doublet remains limited but is expanding (Table 1). Hane and colleagues described 2 patients with relapsed or refractory DLBCL-type RT who achieved rapid, durable complete responses with epcoritamab plus pirtobrutinib; neither was a candidate for CAR T-cell therapy (14). A subsequent multicenter real-world series reported meaningful activity with epcoritamab in high-risk RT, including complete remissions and successful bridging to allogeneic transplantation in selected patients (13). The present observation adds specifically documented activity after liso-cel relapse. A biologic rationale for concurrent BTK inhibition is supported by *ex vivo* CLL studies in which epcoritamab-mediated T-cell cytotoxicity was enhanced during BTK inhibitor exposure, including in samples from patients with progressive disease; whether this effect is equivalent with noncovalent BTK inhibition remains unknown (16). Importantly, the strategy is now being evaluated prospectively in the randomized phase 3 ALLG CLL10/CLLRT2 trial comparing epcoritamab plus pirtobrutinib with R-(mini)-CHOP in previously untreated RT (15).

**Table 1:** Selected clinical evidence involving epcoritamab in Richter transformation.

Study/evidence	Regimen and population	Key findings	Relevance
Kater et al., EPCORE CLL-1 (9)	Epcoritamab monotherapy; phase 1b/2; 42 patients with DLBCL-type RT	ORR 47.6% overall; 57.1% in first-line RT and 38.1% in second/later lines; median follow-up 22.9 months	Prospective proof of activity for epcoritamab in RT
Zeremski et al. (13)	Epcoritamab; multicenter real-world RT case series	Meaningful clinical activity, including complete remissions; selected patients bridged to allogeneic transplantation	Supports effectiveness outside a clinical trial
Hane et al. (14)	Epcoritamab plus pirtobrutinib; 2 relapsed/refractory DLBCL-type RT cases	Rapid durable complete responses; neither patient was a CAR T-cell candidate	First detailed published experience with the doublet
ALLG CLL10/CLLRT2 (15)	Epcoritamab plus pirtobrutinib vs. R-(mini)-CHOP; randomized phase 3; planned *n* = 116	Primary end point: PFS; no efficacy results available	Prospective validation of the combination
Present case	Epcoritamab plus pirtobrutinib after liso-cel relapse	Complete nodal resolution by 3 months; 2 new bone lesions at 3 months resolved by 10 cycles (Deauville 4→2); ongoing clinical remission	Extends doublet experience to post-CAR T-cell relapse

CAR, chimeric antigen receptor; DLBCL, diffuse large B-cell lymphoma; ORR, overall response rate; PFS, progression-free survival; RT, Richter transformation.

### 3.1 Response to a T-cell-engaging bispecific antibody after CAR T-cell failure

Beyond this pharmacodynamic interaction, the achievement of a deep response to a CD3xCD20 bispecific antibody after failure of CD19-directed CAR T-cell therapy raises a broader biological question: could prior CAR T-cell exposure influence subsequent responses to T-cell-engaging therapy? CAR T-cell and bispecific antibody approaches both depend on autologous T-cell function but engage the immune system through mechanistically distinct routes. CAR T-cell therapy relies on *ex vivo*-expanded, antigen-specific effector cells that are re-infused into the patient, whereas epcoritamab depends on the patient's own circulating and tissue-resident polyclonal T-cell repertoire being redirected *in vivo* through CD3 engagement. It is therefore biologically plausible that endogenous T cells not directly involved in prior CAR T-cell manufacturing, lymphodepletion, or antigen-driven exhaustion retain sufficient functional capacity to mediate a bispecific-driven response even after CAR T-cell failure, and that failure of the CAR T-cell product itself (for example, due to antigen loss, CAR T-cell exhaustion, or a hostile tumor microenvironment) does not necessarily preclude a subsequent T-cell-engaging response achieved through a different mechanism and a different target antigen (CD20, as opposed to the CD19 target of the prior CAR T-cell product). This concept remains largely theoretical: the evidence for this specific treatment sequence (bispecific antibody after CAR T-cell relapse) is limited to small case series (13, 14) and the present report, and the relative contributions of host T-cell recovery, target-antigen retention, and concurrent BTK inhibition to the observed response cannot be disentangled from a single case.

### 3.2 Response assessment and PET/CT interpretation

The interim PET/CT at 3 months illustrates a broader interpretive challenge in this setting. Two new FDG-avid osteolytic lesions appeared in the right humeral shaft and lower sacral segment at a time when all other nodal and splenic disease had resolved completely. Under a strict application of the Lugano classification, new FDG-avid lesions can be sufficient to define progressive metabolic disease (8). However, the Lugano framework explicitly allows for an indeterminate or equivocal designation when new or persistent FDG uptake cannot be confidently attributed to lymphoma, particularly in the presence of a markedly favorable, discordant response elsewhere (8, 17). In this patient, several features supported an indeterminate rather than a progressive interpretation: the extensive baseline osteolytic disease with cortical erosion and fracture risk created a substantial pretest likelihood of concurrent reactive bone remodeling and healing-related FDG uptake at previously involved sites; the new lesions were not biopsied, so a neoplastic origin was never histologically confirmed; and the marked discordance between the near-complete nodal response and only 2 new, isolated bone lesions favored a localized reparative or inflammatory process over systemic progression. The subsequent resolution of both lesions by cycle 10 is reassuring and, in retrospect, consistent with a transient, non-malignant process.

This case also highlights a more general limitation of applying conventional Lugano and Deauville criteria (8), which were developed largely in the chemoimmunotherapy era, to patients treated with cellular and T-cell-engaging immunotherapies. Existing literature on CAR T-cell therapy describes pseudoprogression and immune-related inflammatory FDG uptake as recognized, if incompletely understood, phenomena that can mimic true progression on early post-treatment imaging (18, 19). Although this literature has been generated predominantly in the CAR T-cell setting, analogous immune-activation-related uptake is biologically plausible during subsequent T-cell-engaging therapy such as epcoritamab, given the shared dependence on T-cell activation and local inflammatory infiltration. Semiquantitative PET parameters (SUVmax, metabolic tumor volume, total lesion glycolysis) and structured response frameworks developed in the CAR T-cell setting may help refine interpretation in this population but have not been validated for bispecific antibody therapy or for combination cellular/T-cell-engaging sequences (17, 19–21). We therefore consider it important to explicitly include pseudoprogression and immune-related inflammatory uptake in the differential diagnosis of transient new FDG-avid lesions occurring during or after cellular and T-cell-engaging therapies, even when, as in this patient, a specific mechanism cannot be confirmed.

Molecular response assessment could have helped resolve this interpretive uncertainty. Circulating tumor DNA (ctDNA)-based minimal residual disease assessment is increasingly used to complement or refine PET/CT-based response assessment in DLBCL and after CAR T-cell therapy, with the potential to distinguish residual or recurrent lymphoma from inflammatory or reparative FDG uptake when imaging findings are equivocal (22). No molecular assessment was performed in this patient, which we now acknowledge explicitly as a limitation. A multimodal approach integrating serial PET/CT, clinical assessment, and molecular biomarkers such as ctDNA, where available, is likely to become increasingly relevant for response assessment following cellular and T-cell-engaging therapies, including in RT, and represents an important direction for future prospective study.

### 3.3 Strengths and limitations

This report is strengthened by serial PET/CT imaging that documents a progressively deepening metabolic response over four scans and by detailed, prospectively collected tolerability data in a nonagenarian-adjacent patient. It is limited by its single-patient design; a follow-up duration that, despite confirmation of a deepening metabolic response on a second PET/CT, remains relatively short relative to the natural history of RT relapse; inability to attribute benefit to either component of the doublet; absent molecular clonality testing linking the CLL and RT clones; the absence of a formal geriatric or frailty assessment beyond ECOG performance status; the lack of molecular (ctDNA-based) response assessment, which could have helped clarify the nature of the interim FDG-avid bone lesions; and residual uncertainty as to whether these transient lesions represented treatment-related inflammatory activity, reparative bone remodeling, pseudoprogression, or truly indeterminate low-volume disease, since they were not biopsied.

## 4 Conclusion

This case demonstrates that epcoritamab plus pirtobrutinib can produce a deep, progressively deepening metabolic response after CAR T-cell relapse in RT, a setting with no established standard of care. The depth and durability of disease control after post-CAR T-cell relapse support further evaluation of epcoritamab plus pirtobrutinib as a chemotherapy-free salvage strategy for selected patients with multiply relapsed RT. Larger, prospective series with longer follow-up ideally incorporating multimodal response assessment that combines serial PET/CT with molecular biomarkers are needed to define its durability and optimal place in the post-CAR T-cell treatment sequence.

## 5 Patient perspective

The patient and his family reported a clear improvement in his symptoms after starting treatment, particularly in the headaches and bony pain that had been troublesome during disease progression. They were also very satisfied with the tolerability and convenience of the treatment. The subcutaneous administration was considered easy and comfortable, and the oral medication was simple to take at home. Importantly, they did not notice any significant treatment-related side effects, and the therapy did not adversely affect his daily activities, independence, or overall quality of life. Both the patient and his family expressed satisfaction with the clinical response and the ease with which the treatment could be incorporated into his usual daily routine.

## Data Availability

The raw data supporting the conclusions of this article will be made available by the authors, without undue reservation.
